# An Immunoperoxidase Monolayer Assay (IPMA) for the detection of lumpy skin disease antibodies

**DOI:** 10.1016/j.jviromet.2019.113800

**Published:** 2020-03

**Authors:** Andy Haegeman, Ilse De Leeuw, Laurent Mostin, Willem Van Campe, Laetitia Aerts, Maria Vastag, Kris De Clercq

**Affiliations:** a Sciensano, Infectious Diseases in Animals, Exotic and Particular Diseases, Groeselenberg 99, B-1180, Brussels, Belgium; b Sciensano, Experimental Center Machelen, Kerklaan 68, B-1830, Machelen, Belgium; c EURL for Diseases Caused by Capripox Viruses, Sciensano, Groeselenberg 99, B-1180, Brussels, Belgium

**Keywords:** IPMA, LSDV, Antibodies

## Abstract

During this study a new Immunoperoxidase Monolayer Assay (IPMA) was developed for the detection of antibodies against lumpy skin disease virus (LSDV) in an easy and low tech setting. Using two dilutions (1:50 and 1:300) in a duplicate format, the test was shown to be highly sensitive, specific and repeatable. In comparison to the VNT and a commercial ELISA, the LSDV-IPMA was able to detect the LSDV antibodies earlier in infected, vaccinated and vaccinated/infected animals. The assay is very flexible as it can be easily adapted for the detection of sheeppox or goatpox antibodies and it can be scaled-up to handle medium size sample sets by preparing the IPMA plates in advance. These plates are safe and can be handled in low biosafety level labs.

## Highlights

• A new immunoperoxidase monolayer assay (IPMA) was developed to detect LSDV antibodies.
• The new test is highly specific and sensitive and is suitable for medium throughput.
• LSDV-IPMA detected the antibodies earlier than the VNT and a commercial ELISA.
• The LSDV-IPMA system is easily adapted for SPPV and GPV.

## 1 Introduction

The capripoxvirus genus (Capx) is comprised of sheeppox virus (SPPV), goatpox virus (GTPV) and lumpy skin disease virus (LSDV). These three double stranded DNA viruses of approximately 150 kbp (Tulman et al., 2002), are the causative agents of severe and contagious diseases in sheep, goats and cattle respectively (Babiuk et al., 2008). Although mortality rates of SPPV and GTPV are generally higher than for LSDV, all three diseases have serious socio-economic impact (Bhanuprakash et al., 2006; Tuppurainen and Oura, 2012). The latter is due to the production losses (reduced milk yield and weight gain, increased abortion rates, damage to wool and hides) and loss of traction (in case of LSDV). Their economic impact and distribution warrants the notifiable status of these diseases in the European Union (Council Directive 82/894/EEC, Commission Decision 89/162/EEC) and by the World Organisation for Animal Health (OIE).

During the last decades capripox viruses have displayed an emerging distribution pattern. LSDV originally confined to southern part of Africa has travelled north- and eastward. It reached Egypt in 1988 (Ali et al., 1990) and Israel in 1989 (Yeruham et al., 1995). It has established itself in the Middle East with virus circulations reported since the 1990’s (for example: Kuwait 1991 and 2014, Lebanon 1993, United Arab Emirates 2000, Oman 2010 and Iran 2014). Recently LSD spread to eastern and south-eastern European countries. First cases of LSDV in Turkey were reported in 2013 and it spread to Cyprus in 2014 and to Greece in 2015. The virus travelled northeast through the Caucasus, affecting Azerbaijan (2014), Armenia and the Russian Federation (2015) and Georgia and Kazakhstan (2016). In 2016 it spread through different Balkan countries (i.e. Bulgaria, North Macedonia, Serbia, Kosovo, Albania and Montenegro) (Tasioudi et al., 2016; FAO, 2017).

The availability and quality of diagnostic tools are often a determining factor for the efficacy of disease control, eradication or prevention. During the last years significant advances have been made in the virological part of the diagnostics with the development of capripox specific real-time PCRs with associated high sensitivity and specificity (Balinsky et al., 2008; Bowden et al., 2008; Lamien et al., 2011; Haegeman et al., 2013). In contrast, the serological tools have been addressed less. Although high levels of specificity for capripox can be obtained with the virus neutralisation test (VNT), sensitivity can vary between 70–96% (Sadri et al., 2002; Gari et al., 2008; Babiuk et al., 2009) which could provide problems detecting low levels of antibodies. Only recently, one ELISA kit has been commercialized and although the initial data are promising, its true potential still needs to be evaluated in the field. Several in-house ELISA systems have been developed either based upon the use of peptides (Tian et al., 2010), whole (inactivated) virus (Babuik et al., 2009) or purified / recombinant proteins such as P32 (Bhanot et al., 2009; Bowden et al., 2009). Although these ELISA’s attain high levels of specificity/sensitivity, the dataset for some of these tests is relatively low and need therefore further validation to ascertain their performance characteristics. Coating of plates with protein or whole virus require sufficient amounts of high quality stabilized antigen and the production hereof is challenging and needs sophisticated lab equipment. The accompanying quality controls and biosafety requirements make it less suited to be employed in less equipped laboratories which is an important issue considering the distribution of the capripox viruses. Immunoperoxidase Monolayer Assays (IPMAs) have been developed/compared for a large number of viruses such as swine influenza (Direksin et al., 2002), swine hepatitis E (Liang et al., 2014), porcine circovirus type 2 (PCV2) (Pileri et al., 2014), vaccinia virus (Gerber et al., 2012), African swine fever (Afayoa et al., 2014), etc. The benefits of the test are: the simplicity in execution, the basic equipment requirements and the lack of large amounts of (purified) antigen needed without comprising the sensitivity (Pileri et al., 2014) and specificity. It was therefore the purpose of this study to develop an IPMA capable of detecting LSDV antibodies in a relatively low-tech environment, which is cost-effective and more rapid than the current VNT’s without additional biosafety requirements. In addition, the potential to rapidly adapt the test for detecting sheep and goat pox virus antibodies was analysed.

## 2 Materials and method

### 2.1 Virus and cells

The ovine testis cell line OA3.Ts (ATCC-CRL-6546) was cultured in DMEM supplemented with 10 % foetal calf serum (FCS), fungizone (Fisher Scientific; 1 μg/ml) and gentamycin (VWR; 20 μg/ml).

The LSDV strain Neethling was propagated on OA3.Ts as described by Babiuk et al. (2007). Briefly, a 80–90 % confluent cell culture flask (175 cm^2^) was inoculated with 200 μl LSDV (titer 10^5^/ml TCID_50_) in 20 ml growth medium (DMEM + 2 % FCS + fungizone 1 μg/ml and gentamycin 20 μg/ml) and subsequently incubated for 4 days at 37 °C in the presence of 5 % CO_2_. After a freeze/thaw cycle the supernatant was collected after centrifugation (3000 rpm, 10 min) and stored at liquid nitrogen as individual aliquots in order to minimize the effects of freeze-thaw cycles on virus stability.

Virus titration was performed in 96-wells plates using OA3.Ts cells passage 4 to passage 11. Cells were seeded at 10^4^ cells/well in order to achieve 90 % confluence after 24-hr incubation at 37 °C. The outer wells (column 1 and 12, row A and H) of the plate are not used as a drying effect has been noticed in these wells in the past, influencing cell quality and subsequent staining. Plates were infected with 100 μl of a 10-fold serial dilution of the LSDV with 10 replicates per dilution and incubated for 3 days. Viral plaques were detected by IPMA as described below with the exception that a positive anti-LSDV serum was used as primary antibody diluted 1:400 in dilution buffer (1 % skim milk powder in 1xPBS). The virus titers were calculated using the method of Reed and Muench (1938), where wells displaying 1 or more viral plaques were designated as positive.

The GTPV strain Oman and a LSDV strain Neethling were kindly provided by Dr. E. Tuppurainen, at that time Head of the OIE Reference Center for lumpy skin disease at the Pirbright Institute (United Kingdom). The SPPV strain Bangladesh was kindly provided in 2004 by Dr. P. Kitching, at that time working at National Centre for Foreign Diseases, Winnipeg, Manitoba, Canada in collaboration with Dr. P. Mellor, at that time working at the Pirbright Institute (United Kingdom).

In order to investigate the biosafety aspect of the IPMA plates (see section 3.5) the absence or presence capripox genomes was examined using the real-time PCR as described in Haegeman et al. (2013).

An in-house Belgium orf strain was used for analytical specificity testing of the GTPV-and SPPV-IPMA

### 2.2 Immunoperoxidase monolayer assay (IPMA)

Confluent 96-well plates containing OA3.Ts were prepared. Depending on the purpose the cells were infected with 100 TCID_50_ of LSDV strain Neethling (LSDV-IPMA), SPPV strain Bangladesh (SPPV-IPMA) or GTPV strain Oman (GTPV-IPMA). Following 3 days incubation at 37 °C, the growth medium was removed and subsequently washed twice with 1xPBS (100 μl/well) and dried for 1 h at 37 °C. The plates were frozen at −80 °C for minimum 30 min or overnight. The cells were then fixed with 4 % para-formaldehyde (diluted in PBS, 100 μl/well) for 10 min. The fixative was removed and washed twice with 1xPBS for 5 min. After removal of the PBS, 100 μl was added of a freshly made 30:1 methanol/30 %H_2_O_2_ mixture. After 5 min of incubation at room temperature the cells were washed again twice with 1x PBS.

The test sample was diluted 1:50 and 1:300 in blocking buffer (1 % skim milk powder in 1xPBS) and each dilution was added in duplicate to the wells (50 μl/well) and incubated for 1 h at 37 °C. The solution was removed and the wells were washed twice with PBS containing 0.1 % Tween 20 (PBST). Fifty μl of the secondary antibody, conjugated to horseradish peroxidase, (for LSDV-IPMA: Anti-Bovine IgG-peroxidase, Sigma Aldrich; for SPPV-IPMA: Anti-Sheep IgG-peroxidase, Sigma Aldrich; for GTPV-IPMA: Anti-Goat IgG-peroxidase, Sigma Aldrich) was diluted 1:1000 in blocking buffer and was added to each well. The plates were incubated for 1 h at 37 °C. After a final wash (2 times with PBST), 50 μl per well of a substrate solution (3-amino-9-diethyl-carbazole in 50 mM Na-acetate buffer (pH 5) with 0.05 % hydrogen peroxide) was added to reveal the reaction. The mixture was incubated at room temperature for 15 min. The staining was stopped by eliminating the substrate and adding 100 μl of the Na-acetate buffer. Finally the staining was analysed using an inverted contrast microscope. If an accurate antibody titer is required a 2- (or 10-) fold dilution series of the sample can be used.

In addition to the samples, a LSDV positive and negative serum is added to each plate as controls and points of comparison.

### 2.3 Virus neutralisation

A test serum can either be titrated against a constant titer of capripoxvirus of 100 TCID_50_ (Method 1) or a standard virus strain can be titrated against a constant dilution of test serum in order to calculate a neutralisation index (Method 2). LSDV Neethling, SPPV Bangladesh and GTPV Oman were used as viral strains for respectively LSDV, SPPV and GTPV VNT’s.

Both methods are based upon the OIE Terrestrial Manual Chapter 3.4.12 (LSD) and 3.7.12 (SPP and GP) (OIE, 2018a,b) but were adapted for visualisation by IPMA. The latter was carried out as described with the exception that a LSDV, SPPV or GTPV positive in-house reference serum was used as primary antibody.

#### 2.3.1 Method 1 (VNT1)

Confluent 96 well plates (OA3.Ts) were prepared as described. The serum sample was diluted 1:2 in growth medium and heated at 56 °C for 30 min. Subsequently a 5-fold dilution series was made (range 1:2 to1:156,250) in growth medium. Equal amounts of virus (100 TCID_50_) and diluted sera were mixed and incubated at 37 °C for 1 h. From the serum-virus suspension 100 μl was transferred onto the confluent monolayer. Cell control (100 μl of medium) and virus control (50 μl of 100 TCID_50_ virus + 50 μl medium) were added in 3 duplicates each (column 9 and 10) on each plate. The plates were incubated for 3 days at 37 °C. The plates were stained with the IPMA method as described above with the exception that the positive reference in-house serum in dilution buffer was used as primary antibody.

#### 2.3.2 Method 2 (VNT2)

Test sera including a negative and a positive control are diluted 1:5 in growth medium and heated at 56 °C for 30 min. The LSDV Neethling strain was diluted in growth medium to give a dilution series of log_10_ 5.0; 4.0; 3.5; 3; 2.5; 2.0; 1.5 TCID_50_ per ml. Equal amounts (50 μl) of test sera, positive and negative control are mixed with the virus dilution series and incubated at 37C° for 1 h (row A to G). In the wells of row H only 100 μl of cell culture medium was added (cell controls). Subsequently, 100 μl of an OA3.Ts cell suspension (10^5^ cells/ml) is added to all wells. The microtiter plates were incubated at 37 °C for 4 days and stained with the method IPMA as described. The neutralisation index (NI) was calculated as the log titer difference between the titer of the virus in the negative serum and in the test serum. A NI of > = 1.5 is considered as positive.

### 2.4 Commercial ELISA

The ID Screen® CPV Double Antigen (IDVET, Montpellier, France) was used according to the manufacturer’s instructions.

### 2.5 Virus isolation from LSDV IPMA plates

Following the IPMA protocol, after the treatment with methanol and H_2_O_2_ and elimination of the PBS of the subsequent washing steps, the cells were collected by scraping all the wells of one plate. The cells were then suspended in 2 ml of sterile water. Three confluent cell culture flask (OA3.Ts; 25cm^2^) were inoculated with 500 μl of the cell suspension + 500 μl growth medium. Additional growth medium was added after 1 h (2 ml) and 24 h (7 ml). The flask were left to incubate at 37 °C for 2 weeks after which the medium was collected for: 1) real-time PCR testing and 2) for additional passages (n = 2). The cells were tested for the presence of LSDV using IPMA as described above with the exception that: 1) 2 ml PBST was used for each washing step and 2) 1 ml of the primary (in-house positive LSDV serum), secondary Anti-Bovine IgG-peroxidase antibody and coloring substrate was applied for visualization.

### 2.6 Samples

#### 2.6.1 LSDV and SPPV cross reactivity

Exploratory experiments were carried out to investigate the importance of LSDV or SPPV infected plates in the evaluation of the LSDV- and SPPV-IPMA. For this purpose 3 SPPV and one LSDV positive serum, obtained from animal experiments, were analysed on LSDV and SPPV infected plates. Each of the 4 sera were tested on 2 different days with 10 repeats on day 1 and three on day 2. The capripox positive status of these 4 samples was confirmed with the commercial Elisa described in section 2.4. In addition, a kinetic time series, consisting of 13 sera samples, obtained from an animal experimentally infected with LSDV was analysed on LSDV and SPPV infected plates.

#### 2.6.2 Diagnostic and analytical specificity (DSp and ASp)

For diagnostic specificity of the LSDV-IPMA, bovine sera (n = 116) were collected during various animal trials in our animal facilities before vaccination/infection was carried out. The animals were obtained from Belgium farms and can be considered capripox negative as Belgium has a capripox free status. In addition, field sera (n = 28) were also obtained from various veterinarians taken at various Belgian farms and have a similar status. Furthermore, in-house sera, positive for antibodies against bluetongue virus (BTV) (n = 18) or foot and mouth disease virus (FMDV) (n = 19) were included for analytical specificity testing

For DSp analysis of SPPV- and GTPV-IPMA, sheep (n = 11) and goat (n = 12) sera were collected in our animal facilities before any manipulation had been carried out. Additional 28 sheep sera were collected from the field on Belgian farms. As Belgian is free of sheeppox and goatpox all the sera derived from Belgian can be considered as being capripox negative.

#### 2.6.3 Diagnostic sensitivity (DSe)

One of the objectives was to study the DSe at two different points in time after infection. Bovine serum samples (n = 30) were analyzed at 8 and 15 days post infection (dpi). The samples were considered to be positive as they originated from animals which: 1) were experimentally infected with LSDV under controlled conditions during various animal trials; 2) all animals displayed a LSDV associated fever peak around 4/9 dpi following challenge.

Field sera from LSD-vaccinated cattle (n = 87), collected 1 month after vaccination, were similarly used to investigate DSe of the LSDV-IPMA. The status of the animals was considered to be positive as they were vaccinated under controlled conditions.

Field sera obtained from 41 clinically-affected sheep in 19 flocks located within four provinces in the eastern region of Morocco (Haegeman et al., 2016) were used to evaluate the DSe of the SPPV-IPMA. The status of animals was considered positive as the animals were clinically affected and tested positive in either blood, tissue and/or swabs for SPPV.

#### 2.6.4 Kinetics: comparison between IPMA, VNT1 and VNT2

In addition to the diagnostic sensitivity, the kinetics of the antibody response was analyzed using consecutive serum samples that were collected during animal experiments from: 1) cattle (n = 8), sheep (n = 7) and goats (n = 7) experimentally infected with LSDV (Neethling), SPPV (field isolate from Morocco) and GTPV (Oman), respectively; 2) cattle (n = 7) which were vaccinated with a LSD-based live attenuated vaccine; 3) sheep (n = 4) and goats (n = 2) which became infected by contact transmission, as demonstrated by the development of fever, noduli, viremia (detected using the real-PCR of Haegeman et al., 2013), during the course of the trial and therefore represent natural occurring infection (referred to as Exp3: G4 and G5, S4 to S7, Tables 1,2).

**Table 1:** LSDV-IPMA titration results of R6F45, expressed as the logarithmic reciprocal value of the dilution, using plates stored at different conditions (temperature and duration).

Temp / days	0	10	17	31	38	59
4 °C	3,9	4,1	4,4	3,3	2,2	2,2
−20 °C		3,7	4,1	4,1	4,1	4,2
−80 °C		3,8	4,2	4	4,1	4,1

**Table 2:** Comparative data VNT method 1/VNT method 2/SPPV-IPMA on sera of infected sheep. (a): end of experiment; NA: Not Available; -: not tested; N: Negative; P: Positive.

	Exp 1		Exp 2		Exp 3						
dpi	S1	S2	S1	S2	S1	S2	S3	S4	S5	S6	S7
0	N/-/-	N/-/-	N/-/-	N/-/-	N/-/-	N/-/-	N/-/-	N/-/-	N/-/-	N/-/-	N/-/-
2	N/-/-	N/-/-	N/-/-	N/-/-	N/-/-	N/-/-	N/-/-	N/-/-	N/-/-	N/-/-	N/-/-
4	N/N/N	N/N/N	N/N/N	N/N/N	N/N/N	N/N/N	N/N/N	N/-/-	N/-/-	N/-/-	N/-/-
7	P/N/P	N/N/P	P/N/P	P/N/P	N/N/P	P/N/P	N/N/N	N/-/-	N/-/-	N/-/-	N/-/-
9	P/N/P	P/N/P	P/P/P	P/P/P	N/N/P	P/N/P (a)	N/N/P	N/-/-	N/-/-	N/-/-	N/-/-
10	NA	P/N/P (a)	P/P/P (a)	P/P/P (a)	NA		P/N/P (a)	NA	NA	NA	NA
11	P/P/P				P/N/P			N/-/-	N/-/-	N/-/-	N/-/-
14	P/P/P				P/P/P (a)			N/-/-	N/-/-	N/N/N	N/-/-
16	P/P/P							N/-/-	N/-/-	N/N/P	N/-/-
17	P/P/P (a)							N/-/-	N/-/-	N/N/N	N/N/N
18								N/N/N	N/N/N	P/N/P	N/N/N
21								N/N/N	N/N/N	N/N/P	N/N/N
25								N/N/P	P/N/N	P/N/P	P/N/N
28								P/N/P	P/N/P	P/P/P	P/N/P
29								P/N/P (a)	P/P/P (a)	P/N/P (a)	P/N/P (a)

#### 2.6.5 Kinetics: comparison between LSDV-IPMA and ELISA

Consecutive serum samples collected during animal experiments from infected cattle (n = 6) and from infected /vaccinated cattle (n = 6) were used to compare the antibody response over time.

#### 2.6.6 Repeatability and storage capacity

Two strong LSDV positive (R6F45 and R6F35) and 1 weak SPPV (S6F29) sera were selected from LSDV and SPPV infection trials for the evaluation of the repeatability. One of these sera, R6F45, was further used to create a panel for blind testing. This panel consisted of 4 negative samples, R6F45 (undiluted) in duplicate, R6F45 (1:2 diluted), R6F45 (1:4 diluted) in duplicate and R6F45 (1:10 diluted).

In order to evaluate the possibility to store the plates following methanol/H_2_O_2_ treatment and subsequent washing, freshly prepared LSDV-IPMA plates were placed at 4 °C, -20° and −80 °C and stored for 0, 17, 31, 38 and 59 days before testing with R6F45. Similar to the repeatability testing, a 2-fold dilution was used of R6F45. The storage ability was also evaluated using a LSDV positive reference serum from the Pirbright Institute (UK).

## 3 Results

### 3.1 LSDV and SPPV cross reactivity

Cross reactivity was seen for the bovine sera as the 13 samples in the time series as well as the sera included in the repeat testing were scored positive independently of the plates were infected with LSDV or SPPV. However, it was noted that for the 10 of 13 samples in the kinetic time series the coloration (intensity and number of positive foci) was weaker in the 1/300 dilution when SPPV infected plates were used compared to LSDV plates. Furthermore, 2 of the 3 ovine sera scored negative on LSDV plates while being positive on SPPV plates and this on both days and in all repeats. In contrast the third ovine scored equally positive on LSDV and SPPV plate.

### 3.2 Diagnostic and analytical specificity of the LSDV-IPMA

**Fig. 1:**
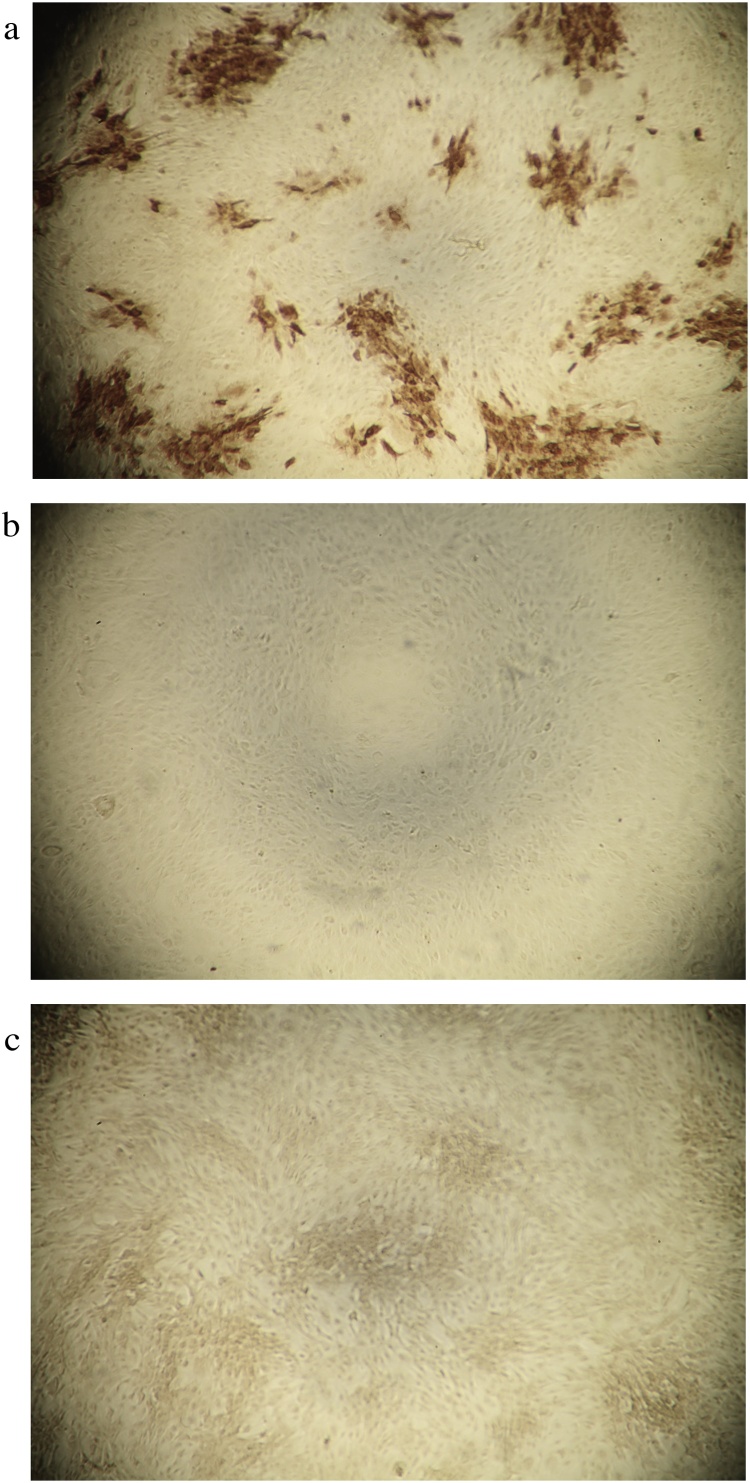
Examples of IPMA staining in a 96 well plate; A: positive staining (LSDV positive clusters); B: negative staining; C: negative staining with increased background.

In total 116 untreated cattle were sampled in our animal facilities and tested with the LSDV- IPMA. No coloration of the cells was observed and all were correctly scored negative. Examples of positive and negative staining can be seen in Fig. 1A and B. As these sera were taken, transported and stored under ideal circumstances, sera received from veterinarians in the field (cattle, n = 28) were analyzed as well. Similar to the previous samples, none of the wells were stained and all samples were scored negative.

To evaluate the effect of other non-capripox antibodies in the sera, strongly positive sera for BT and FMD were used as primary antibodies in the LSDV-IPMA as these diseases are often endemic in countries where LSD is present (even in SE-Europe for BT). In total 18 BTV positive sheep sera and 19 FMD positive bovine sera were analysed and were scored negative. As the highest interference is to be expected for the highest concentration only the smallest dilution of the LSDV-IPMA (1:50) was evaluated. Two randomly selected BTV positive and 2 FMD positive sera were analysed in depth (24 repetitions). No coloration was obtained for the FMD serum, while 2 doubtful results were obtained for one of the BTV sera. In the latter some coloration was obtained in only one of the duplicates in two of the 24 replicates.

### 3.3 Diagnostic sensitivity of the LSDV-IPMA

The diagnostic sensitivity for the LSDV-IPMA, VNT1, VNT2 and ELISA was determined at 8 and 15 dpi. Analysing 30 serum samples from infected bovines resulted in a sensitivity (5 % error with a confidence of 99 %) at 8 dpi of 56.7 % for LSDV-IPMA while this was 0 % for the other three assays. At 15 dpi the sensitivity was 100 % for LSDV-IPMA while this was 63.3 %, 60 % and 16.7 % for VNT1, VNT2 and ELISA respectively.

When using the 87 field sera, taken at 30 dpv, the DSe of LSDV-IPMA was found to be 41.8 % while this was 50.5 % for the ELISA (5 % error with a confidence of 95 %). It needs mentioning that the sample quality was less ideal with clear hemolysis in a great number of samples resulting in increased background staining (Fig. 1c). Eighteen samples (19.8 %) scored doubtful in the LSDV-IPMA.

### 3.4 Kinetics: comparison between LSDV-IPMA, VNT1, VNT2 and ELISA

**Fig. 2:**
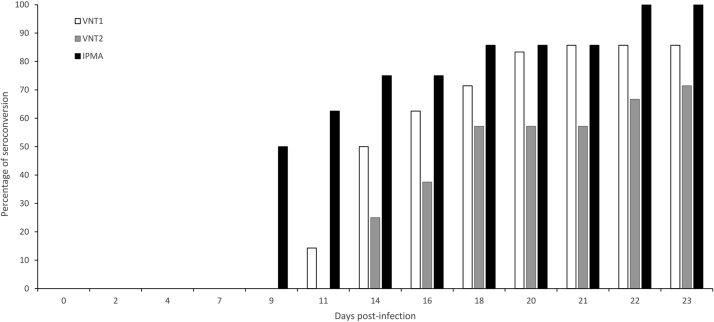
Kinetics: percentage seroconversion by VNT1, VNT2 and IPMA with sera from cattle infected with LSDV (n = 8).

As the DSe data suggests that antibodies are earlier detected by LSDV-IPMA, consecutive sera samples obtained from infected cattle (n = 8) were analysed with LSDV-IPMA, VNT1 and VNT2. A clear difference was observed between the 3 methods regarding the onset of seroconversion. Antibodies were detected earlier in 100 % of the animals with the LSDV-IPMA compared to VNT1 and VNT2 (Fig. 2). The difference in the onset detected varied between 2 and 9 days with 2 outliers. In one animal, the detection of onset of seroconversion differed with 18 days and in a second animal antibodies were not detected with VNT1 and VNT2 before the end of the trial (45 dpi) while the LSDV-IPMA became positive on 23 dpi.

**Fig. 3:**
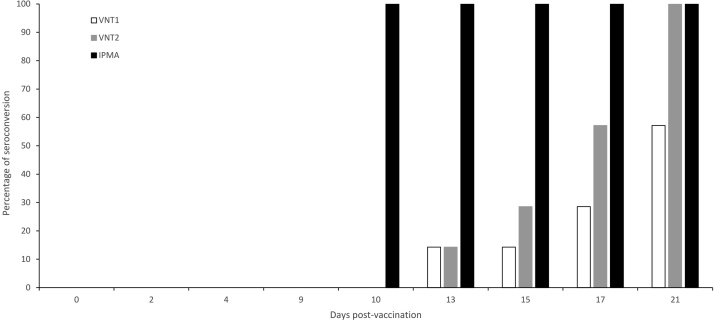
Kinetics: percentage seroconversion by VNT 1, VNT2 and IPMA with sera from cattle vaccinated against LSD (n = 7).

As the antibody kinetics can differ between infection and vaccination, consecutive serum samples were also collected from cattle (n = 7) vaccinated against LSD. These sera were similarly analysed and compared with LSDV-IPMA, VNT1 and VNT2. Similar to infected cattle, the antibodies were earlier detected in vaccinated cattle with the LSDV-IPMA compared to the VNT’s, namely 3–11 days. In 3 animals VNT1 was not able to detect the antibodies before challenge (which was 21 dpv) (Fig. 3).

**Fig. 4:**
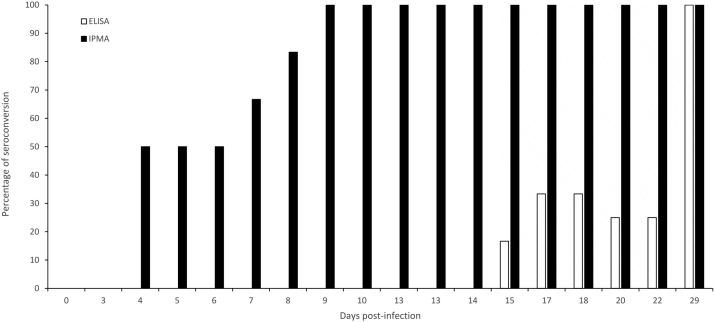
Kinetics: percentage seroconversion by ELISA and IPMA with sera from cattle infected with LSDV (n = 6).

**Fig. 5:**
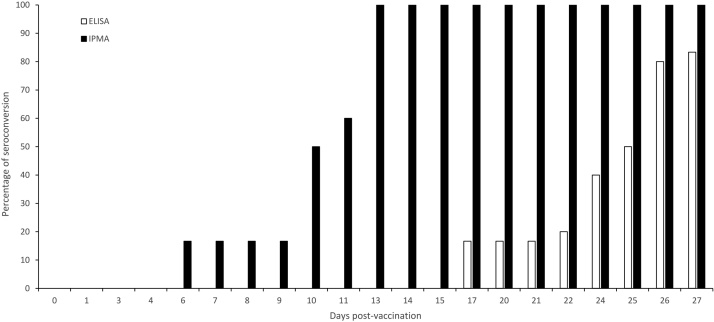
Kinetics: percentage seroconversion by ELISA and IPMA with sera from LSD vaccinated\infected cattle(n = 6). Animals were challenged at 21 Dpv.

A similar comparison was made between LSDV-IPMA and ELISA by using sequential serum samples obtained from experimentally infected (n = 6) and vaccinated\infected animals (n = 6). The LSDV-IPMA detects LSDV antibodies much earlier (7–17 days) than the commercial ELISA in infected as well as in vaccinated/infected animals (Fig. 4, Fig. 5).

### 3.5 Repeatability of the LSDV-IPMA

In order to determine the repeatability, a 2-fold dilution series of two positive LSDV sera (R6F45 and R6F35) were made and tested on 3 different days. The R6F45 serum was tested in triplicate on each day (9 assays) while R6F35 was tested in triplicate on day 1 and 3 and only once on day 2 (7 assays). The obtained titer, expressed as the logarithmic reciprocal value of the dilution, varied between 3.8 and 4.2 for R6F45 and 3.8 and 4.4 for R6F35. The variation corresponded to 1.5–2 dilutions and was seen for plates tested on the same day as well as for plates tested on all three days.

The repeatability testing panel was analysed “blind” by 4 different technicians on 4 different days. The plates were read on the day of coloring (D0) and 1 day later (D1). The negative, the pure and the 1:2 dilution samples were correctly scored by all 4 technicians on D0 and D1. The cut-off samples (1:4 and 1:10) were doubtful on D0 but scored positive on D1. The latter indicates that reading of the plates on D1 can be beneficiary in case of weak positive sera.

### 3.6 Storage and biosafety

The titer of R6F45 using freshly prepared plates at the start of the storage experiment (0 days of extra storage) was 3.9. This falls within the variation seen for this serum sample during the repeatability testing, which was 3.8 to 4.2. The plates kept at 4 °C could be stored up to 17 days without problems after which the titer dropped very quickly. Although the sample remained positive (titer 2.1) on day 38 and 59, the coloration was very weak and difficult to read/interpret. In contrast, plates stored at −20 °C and −80 °C remained usable up to 59 days as the titers remained very similar and within the variation seen during the repeatability testing. The data is summarized in Table 1. The impact of storage was also evaluated using the repeatability testing panel. During the “blind” testing 2 technicians received 2 plates each. One of the plates was stored for 59 days while the other was freshly made. No information about the plates was given prior to analysis. All samples of the panel were scored correctly and no difference was seen in scoring. Similarly, a LSDV reference serum from the Pirbright Institute was titrated on a freshly made plate and a plate stored for one month at −20 °C. The titer obtained was identical for both, namely 3.6.

The biosafety implications of using and storing LSDV infected plates were studied by collecting the cellular material present in the wells after the PBS washes following methanol/H_2_O_2_ treatment. The collected material was used to inoculate small cultures flasks in triplicate. Two subsequent passages were carried out on each of the triplicates. No virus could be isolated from all three passages from any of the culture flasks. Cell culture supernatants were collected after each of the isolations and tested for the presence of capripox genome by real-time PCR. Only the cell culture supernatant of the first isolation was borderline positive in 2 of 3 replicates (Ct 39.8 and 39.7) while both other passages were negative for all three replicates.

### 3.7 Adaptation for detecting SPPV and GTPV antibodies (SPPV-IPMA, GTPV-IPMA)

To evaluate potential false positive results a confluent 96-well plate was infected with orf virus (ORFV), a parapoxvirus inducing similar clinical aspects in sheep and goats as capripox virus infections. One SPPV and 2 GTPV positive sera were used as primary antibody and each had 6 replicates. No positive results were obtained.

To evaluate the diagnostic specificity of the SPPV- and GTPV-IPMA, 11 sheep and 12 goats were sampled in our animal facilities and another 28 sheep sera were obtained from the field. Identical to the LSDV-IPMA no coloration of the cells was observed and samples were therefore all scored negative. Using the 41 Moroccan field samples, a DSe of 82,9 %, 75.6 % and 87.8 % was obtained (5 % error with a confidence of 98 %) for the VNT1, VNT2 and the SPPV-IPMA respectively. In view of the fact that a difference was seen in the early detection between the LSDV-IPMA and the other assays, consecutive samples were analysed from sheep (n = 7 + 4) and goat (n = 7 + 2). A similar trend to the LSDV data was seen but was less pronounced than in cattle. The IPMA detected antibodies earlier in 45.5 % (sheep) / 55.6 % (goats) of the animals compared to VNT1 and in 100 % (sheep) / 77.8 % (goats) compared to VNT2. In 18 % and 11.1 % of the sheep and goats, respectively, antibodies were earlier detected with VNT1 than with IPMA. Detection of antibodies with VNT2 superseded that of the GTPV-IPMA only in 1 goat ( = 11.1 %). The data were summarized in Table 2 (sheep) and Table 3 (goats). The difference in detection of the onset was smaller than in cattle and varied between 1–9 days in sheep and around 2 days in goats.

**Table 3:** Comparative data: VNT method 1 / VNT method 2 /GTPV-IPMA on sera of infected goats. (a): end of experiment; NA: Not Available; -: not tested; N: Negative; P: Positive.

	Exp 1		Exp 2		Exp 3				
dpi	G1	G2	G1	G2	G1	G2	G3	G4	G5
0	N/-/-	N/-/-	N/-/-	N/-/-	N/-/-	N/-/-	N/-/-	N/-/-	N/-/-
2	N/-/-	N/-/N	N/-/-	N/-/N	N/-/-	N/-/-	N/-/-	N/-/-	N/-/-
4	NA	NA	N/-/N	N/-/N	N/-/-	N/-/-	N/-/-	N/-/-	N/-/-
5	N/-/-	N/N/N	NA	NA	NA	N/-/-	NA	NA	NA
7	N/-/-	N/N/P	N/-/N	P/-/P	N/-/-	N/-/-	N/-/-	N/-/-	N/-/-
9	N/N/P (a)	P/N/P	N/N/P (a)	P/N/P(a)	N/N/N	N/-/-	N/-/-	N/-/-	N/-/-
11		NA			P/P/P (a)	N/-/-	N/-/-	N/-/-	N/-/-
12		P/N/P (a)				NA	NA	NA	NA
18						N/-/-	N/-/N	N/-/-	N/-/-
21						N/-/-	N/-/N	N/-/-	N/-/-
23						N/-/-	N/N/P	N/-/-	N/N/N
25						N/-/-	P/P/P (a)	N/-/N	N/N/N
28						N/-/-		N/N/P (a)	P/N/P (a)
37						N/N/N			
38						NA			
39						N/N/N			
42						P/N/N			
44						P/P/N			
46						N/N/N			

The repeatability of the SPPV-IPMA was tested using a positive sheep serum S6F29. The latter was tested on three separate days either single (day 1) or in duplicate (day 2 and 3). The titer of S6F29 varied between 2.9 and 3.2. This observed variation corresponded to 1 dilution and was seen within the plates tested on the same day as well as in the overall variation between all three days.

## 4 Discussion

Serological screening of animal populations is a cornerstone of any surveillance, control/eradication strategy. This could be even more prominent in diseases where the clinical picture can be variable and viremia low and/or very short such as for lumpy skin disease (Tuppurainen et al., 2005; Babiuk et al., 2008). The need for serological tools which can be applied in low-tech circumstances was addressed in this study by the development and evaluation of a LSDV-IPMA. The new LSDV-IPMA had a high DSp and was able to detect antibodies earlier compared to the VNT1, VNT2 and ELISA. This can be seen in the comparison of the DSe’s of all 4 assays at 8 and 15 dpi. The ability of the LSDV-IPMA to detect antibodies earlier than the VNT’s and the ELISA, as demonstrated by the DSe, was confirmed by studying the antibody response kinetics in infected as well as in vaccinated cattle. The capacity of the developed LSDV-IPMA to detect all types of antibodies and not solely neutralizing ones could explain the increased sensitivity, compared to the VNT’s, observed in this study (Gerber et al., 2012). When comparing to the commercial ELISA, the difference in affinity for different Ig isotypes, especially IgM versus IgG, between both methods could be an explanation. This difference in DSe disappears when samples are taken at a later stage of the infection or vaccination due to the rise in antibody titers in relation to the time point of infection or vaccination.

In this study the DSe of the ELISA was a bit higher than the DSe of the LSDV-IPMA with the field sera. This was most likely due to the quality of the received samples. Hemolysis was observed in a large number of samples which resulted in a high number of doubtful results with LSDV-IPMA. Low sample quality increases the nonspecific cross-reactions which in turn results in more significant background coloration. This complicates interpretation without a negative sample reference of the same quality. Compared to the ELISA, where no such problems were observed, the LSDV-IPMA seems to be more sensitive to hemolysis or others factors influencing sample quality. Cross-reactivity has been reported in the past with immune-based detection systems (Davies and Otema, 1981; Gari et al., 2008). Diluting the sample 1:25 (Gari et al., 2008) or 1:40 (Milovanović et al., 2019) has been proposed to decrease the impact of cross-reaction. Notwithstanding the fact that the developed LSDV-IPMA uses a 1:50 start dilution, a significant background staining can remain with low quality samples. As no interference was seen with BTV and FMD positive sera in this study, the exact reason of this background staining is unknown but needs be kept in mind for field samples where no “negative” reference serum for the animal is available. The issue of background staining was further addressed in the development of the new LSDV-IPMA by including a second dilution, namely 1:300. A sample with a weak and unclear staining in the 1:50 only (1:300 is negative) will be classified as doubtful if no negative reference is available for the animal. Inclusion of the 1:300 has also a second advantage. Strong positive sera can give an intense and almost uniform staining in the 1:50 dilution that can be misinterpreted as background. A disadvantage of the IPMA, similar to VNT’s, is it labor/time intensiveness and the use of infectious virus. The latter could provoke biosafety issues and require appropriate facilities. For the LSDV-IPMA the labor and time investment lies in the preparation of the infected plates. The sample analysis itself takes no longer than for an ELISA. In this study it is shown that the LSDV-IPMA plates can be stored for up to 2 months without problems at the appropriate temperature. This allows the preparation in advance of a large screening. In addition, no virus could be isolated from the prepared IPMA plates and viral DNA could only be detected in the cellular material of the cells of the first passage and even then at very high Ct values. These detections were caused by the infection of the cells during the initial preparation phase of the IPMA plates. No LSDV genome was detected in the subsequent passages showing that no viral multiplication had taken place. Based on these findings it can be stated that once the plates are made, they can be stored and transferred to lower biosafety labs. Both aspects provide an advantage over VNT’s and alleviate in part the increased labor/time needs compared to ELISA. The overall cost of the IPMA test is comparable to that of the ELISA. The higher personal costs (preparation of the plates and carrying out the test) for the IPMA are compensated by the basic requirements for reagents and materials. The ability to store the IPMA plates makes it possible to prepare them in batches and reduces the necessity to keep the cell line constantly in culture. This further decreases the overall IPMA costs. Nevertheless, for large scale screening, like post-vaccination monitoring, the ELISA remains more suitable.

An additional advantage of the newly developed IPMA is its flexibility. This was demonstrated by the adaptation of the LSDV-IPMA for SPPV and GTPV antibody detection. Although a degree of cross reaction was observed between LSDV and SPPV, this was not complete as demonstrated by the fact that only 1 of the three SPPV positive sera was detected when LSDV IPMA plates were used. The underlying reason is beyond the scope of this study and needs further research, but this shows the need to use SPPV, GTPV and LSDV plates when analyzing ovine, caprine and bovine sera, respectively, for capripox viruses. Although the evaluation of both the SPPV- and GTPV-IPMA during this study was less extensive compared to the LSDV-IPMA, it can be concluded that the SPPV-IPMA and GTPV-IPMA appear to work as well as the LSDV-IPMA. The SPPV-IPMA had a high DSp and no cross-reaction was seen with antibodies against orf virus which is important as orf is endemic in many countries throughout the world. The DSe of SPPV-IPMA was similar to the one of the VNT’s, with a slight preference for the IPMA, using Moroccan field samples. The fact that almost no difference in DSe was seen for those samples was, similar to the bovine field sera, caused by the time point of sample collection in relation to the infection. When the kinetics of the antibody response was compared between the SPPV- and GTPV-IPMA and both the VNT’s, the IPMA’s were able to detect the antibodies earlier in the majority of the animals. However, this was less pronounced than with the LSDV-IPMA for cattle. This could be due to the fact that the clinical disease in sheep and goats was much more severe during the animal trials. This caused a number of animals to be euthanized for ethical reasons relatively quickly making it more difficult to really ascertain the real difference in detection. Secondly, the increased intensity of the disease in sheep and goats could be the result of a higher suppression of the host immunes systems making it more difficult to observe differences in DSe or kinetics.

In conclusion, the newly developed LSDV-IPMA is flexible (can be adapted for SPPV and GTPV), highly sensitive and specific and well suited for confirmatory testing or for small to medium sample sets especially early after infection or vaccination.

## CRediT authorship contribution statement

**Andy Haegeman:** Conceptualization, Investigation, Writing - original draft, Visualization. **Ilse De Leeuw:** Writing - review & editing, Investigation. **Laurent Mostin:** Resources, Supervision. **Willem Van Campe:** Resources, Investigation. **Laetitia Aerts:** Writing - review & editing. **Maria Vastag:** Investigation. **Kris De Clercq:** Writing - review & editing, Supervision, Project administration.

## Declaration of Competing Interest

The authors declare that they have no known competing financial interests or personal relationships that could have appeared to influence the work reported in this paper.
